# Influence of Pollen Nutrition on Honey Bee Health: Do Pollen Quality and Diversity Matter?

**DOI:** 10.1371/journal.pone.0072016

**Published:** 2013-08-05

**Authors:** Garance Di Pasquale, Marion Salignon, Yves Le Conte, Luc P. Belzunces, Axel Decourtye, André Kretzschmar, Séverine Suchail, Jean-Luc Brunet, Cédric Alaux

**Affiliations:** 1 UMT, Protection des Abeilles dans l’Environnement, CS 40509, Avignon, France; 2 ACTA, Site Agroparc, Avignon, France; 3 INRA, UR 406 Abeilles et Environnement, CS 40509, Avignon, France; 4 INRA, UR 546 Biostatistique et Processus Spatiaux, CS 40509, Avignon, France; 5 Université d’Avignon et des pays du Vaucluse, UMR 7263 Institut Méditerranéen de Biodiversité et d’Ecologie, Pôle Agrosciences, Avignon, France; The Australian National University, Australia

## Abstract

Honey bee colonies are highly dependent upon the availability of floral resources from which they get the nutrients (notably pollen) necessary to their development and survival. However, foraging areas are currently affected by the intensification of agriculture and landscape alteration. Bees are therefore confronted to disparities in time and space of floral resource abundance, type and diversity, which might provide inadequate nutrition and endanger colonies. The beneficial influence of pollen availability on bee health is well-established but whether quality and diversity of pollen diets can modify bee health remains largely unknown. We therefore tested the influence of pollen diet quality (different monofloral pollens) and diversity (polyfloral pollen diet) on the physiology of young nurse bees, which have a distinct nutritional physiology (e.g. hypopharyngeal gland development and *vitellogenin* level), and on the tolerance to the microsporidian parasite *Nosema* *ceranae* by measuring bee survival and the activity of different enzymes potentially involved in bee health and defense response (glutathione-S-transferase (detoxification), phenoloxidase (immunity) and alkaline phosphatase (metabolism)). We found that both nurse bee physiology and the tolerance to the parasite were affected by pollen quality. Pollen diet diversity had no effect on the nurse bee physiology and the survival of healthy bees. However, when parasitized, bees fed with the polyfloral blend lived longer than bees fed with monofloral pollens, excepted for the protein-richest monofloral pollen. Furthermore, the survival was positively correlated to alkaline phosphatase activity in healthy bees and to phenoloxydase activities in infected bees. Our results support the idea that both the quality and diversity (in a specific context) of pollen can shape bee physiology and might help to better understand the influence of agriculture and land-use intensification on bee nutrition and health.

## Introduction

By ensuring reproduction of many plants, pollinators, like honey bees, are essential to the functioning of natural and agricultural ecosystems [1–3]. In turn, pollinators benefit from this pollination service by harvesting the nutrients (nectar and pollen) required for their growth and health. For example, in honey bees, floral nectar, containing carbohydrates and stored as honey, is the energetic fuel of individuals, and pollen provides most of the nutrients required for their physiological development [4]. The development and the survival of honey bee colonies are therefore intimately associated with the availability of those environmental nutrients [4–6], which suggests that the alteration of bee foraging areas due to the current intensification of agriculture and landscape changes might provide a deficient nutrition and therefore affect honey bee populations [7,8]. This is further supported by beekeepers, who are ranking poor nutrition and starvation as two of the main reasons for colony losses [9]. Therefore, studying the link between nutrient availability and bee health might help to better understand the current bee losses observed throughout the world [10,11].

Among those flower nutrients, pollen, which is virtually the main source of proteins, amino acids, lipids, starch, sterols, vitamins and minerals [12,13], is a major factor influencing the longevity of individuals [6]. Pollen is also important at the colony level, since it enables the production of jelly by young workers, that is used to feed larvae, the queen, drones and older workers [14,15]. Therefore, a direct consequence of nutritional deficiency (pollen shortage) is a decrease in the colony population [5] and likely a deficient health of individuals, which could also affect the resistance threshold of bees to other stress (pathogens or pesticides) [8,16]. Indeed, pollen intake is known for influencing the physiological metabolism [17,18], immunity [19], the tolerance to pathogens like bacteria [20], virus [21] and microsporidia [22] and reducing the sensitivity to pesticides [23]. However, honey bees rarely face a total lack of pollen in their environment but are rather confronted with variability in time and space of pollen resource abundance, type and diversity. In addition, pollens can differ between floral species regarding their nutritional contents [24–26] suggesting that some are of better quality for bees than others. Therefore, studying the influence of pollen intake on bee health requires also taking into account the quality and diversity of pollen diets. Despite some studies showed that pollen quality can affect the longevity of bees [27–30] and the hypopharyngeal gland development [29,31] and, more recently, that pollen diversity might improve some immune functions [19], our knowledge of the influence of quality and diversity of pollen diets on bee health is rather limited.

To improve our knowledge on this topic, the influence of pollen diet quality and diversity was tested on nurse physiology and the tolerance to a parasite. Since pollen is essentially consumed by young nurse bees, they have a very specific nutritional physiology with large lipid and protein stores (see 32,33 for reviews). Notably, pollen intake enables the development of their hypopharyngeal glands, where digested pollen nutrients are used to produce jelly, a proteinaceous glandular secretion shared with nestmates [14,15]. Nurse bee physiology was thus assessed by determining the development of the hypopharyngeal glands but also the gene expression level of *vitellogenin*, which is highly expressed in nurses as compared to foragers [34], and encodes a major protein produced in the fat body and used for jelly production [35]. This gene, that can be nutritionally regulated [17,18], also slows down aging [36] and is involved in the regulation of cellular immune functions [37]. We included the analysis of the gene *transferrin*, an iron transport protein also produced in the fat body, and involved in ovary development [38–40] and immune response [41], like *vitellogenin*. However, it is not known whether it is nutritionally regulated, which will be tested through this study. Finally, the tolerance to parasitism was tested using the highly prevalent microsporidia Nosema *ceranae* , a gut parasite that might play a role in colony losses or honey bee weakening [42–45]. For that purpose, we assessed the effects of pollen diet and parasite on bee survival and on physiology by measuring the activity of glutathione-S-transferase (GST), phenoloxidase (PO) and alkaline phosphatase (ALP). GSTs are important in the detoxification of endogenous and exogenous compounds [46] and can be induced in insect gut after bacterial infection, suggesting a protective role against pathogens [47]. In addition, previous studies showed a higher GST activity after *Nosema* infection in bees [48,49]. PO plays an important role in insect immunity by encapsulating pathogens (e.g. bacteria and fungi) and repairing tissues via melanogenesis [50], and ALP, involved in many metabolic processes, is highly expressed in insect gut and plays a pivotal role in intestine health in mammals [51].

## Materials and Methods

### Pollen diet composition and nutritional factors

The effects of pollen quality and diversity were tested by feeding bees with monofloral diets that differed regarding their nutritional properties or a polyfloral diet composed of the different monofloral pollens. Four blends of wild flower pollens with a respective predominance of *Cistus, Erica,* *Castanea* and *Rubus* pollens were purchased fresh from Pollenergie® (France) and stored at -20° C. Pollen pellets were collected from pollen traps at the hive entrance. Monofloral pollen diets of *Cistus, Erica,* *Castanea* and *Rubus* were obtained by sorting by color the pellets of the predominant pollen from each blend. Palynological tests were then performed to validate the genus of each sorted pollen. The polyfloral pollen diet was composed of a mixture of the four monofloral pollens (25% of each according to their weight).

To assess the nutritional quality of each pollen diet, we analyzed their protein, amino acid, lipid and sugar contents, as well as their antioxidant capacities. The protein content was determined by microkjeldahl analysis (N x 6.25) using a Vapodest 45 (Gerhardt) and according to the procedure ISO 5983-2 [52]. Total lipids were analyzed after the disruption of pollen wall using an acid hydrolysis with hydrochloric acid (HCl 6N). Then lipids were extracted with a chloroform/methanol mixture (2:1, v/v) following the method of Folch et al. [53]. The protein and lipid contents were expressed as percent of dry matter, which was determined after drying the pollen for 24 h at 75° C [54]. The nature and the concentrations of amino acids were determined in 20 mg of pollen with the ion-exchange chromatography technique using an automated amino acid analyzer according to the procedure EC 152/2009 [55]. The Oxygen radical absorbance capacity (ORAC) method with AAPH (2,2’-azobis(2-amidino-propane) dihydrochloride) as a free-radical generator was used, as described by Ou et al. [56], to measure the antioxidant capacity in 1 g of each pollen. The antioxidant trolox was used as a standard and thus the data expressed as trolox equivalent. To qualitatively measure sugar contents the pollens were dehydrated for 48 h at 35° C. Thirty mg of pollen were weighed and 1000 μl of Ultrahigh-quality water (18.2 mΩ) were added. The content was passed with a Hamilton syringe through a 0.2 µm filter (Millex LG CI, 0.2 microns; Millipore) and injected into HPAEC Dionex ICS- 3000 equipment. Separation of carbohydrates was carried out on a CarboPac PA-1 guard column (4 x 50 mm) and a CarboPac PA-1 anion-exchange column (4 x 250 mm) after two-fold dilution. The quantitative determination of carbohydrates was carried out by pulsed amperometric detection [57]. The presence of pesticide residues in the different pollen diets was assessed via gas and liquid chromatography with a limit of quantification of 0.01 mg/kg and a limit of detection of 0,005 mg/kg following the AFNOR 15662 procedure [58] (List of analyzed pesticides in Table S1).

### Bee rearing and feeding

To control the pollen intake, the experiments were performed on 1-day-old bees (*Apis mellifera*) reared in cages (10.5 cm x 7.5 cm x 11.5 cm). Age-matched bees were obtained by placing honeycombs containing late-stage pupae into an incubator at 34° C and 50-70% of humidity, and collecting bees that emerged within 10 hours. They originated from three colonies and were mixed before placing them in cages. The caged bees, kept in an incubator (34° C and 50-70% of humidity), were provided *ad libitum* with candy (Apifonda + powdered sugar) and water. Groups of bees were fed with one of the following monofloral pollen diets: *Erica, Cistus, Rubus* or *Castanea* , a mixture of the four pollens (polyfloral diet) or did not receive any pollen. Pollen diets were prepared by mixing pollen with water at the mass ratio of 10/1 (pollen/water) and were freshly prepared and replaced every day for 7 days. To prevent a potential nutritive compensation of bees fed with one of the pollen diet, they were not provided with *ad libitum* pollen but with determined quantity of pollen each day: 4 mg/bee the first two days, 5 mg/bee the next two days, 3 mg/bee the fifth day, and 2 mg/bee the last two days. Those quantities were determined through preliminary experiments and represent the minimal consumption of all pollens on each day; and as previously found pollen consumption varies with age of the bees (increased the first days and then decreased) [4,31]. Using this method, bees were provided with the same quantity of each pollen diet and consumed all of it on each day. Since some bees died during the pollen feeding period (7 days), the pollen quantities were adjusted each day to the number of surviving bees.

### Influence of pollen quality and diversity on bee nurse physiology

Groups of 35 one-day old bees were placed in cages and reared for 7 days with one of the pollen diet. On day 8, they were flash frozen in liquid nitrogen and stored at -80° C for subsequent physiological analyses. The experiment was repeated 14 times per pollen diet.

#### Development of hypopharyngeal glands

The right and left glands form of five bees per cage were dissected on ice in 100 µl of physiological serum (0.9% NaCl). Both glands were slide-mounted and analyzed under an optical microscope coupled to a CF 11 DSP camera (Kappa). The gland development was assessed by measuring the maximum diameter of 15 randomly chosen acini per gland (*n* = 30 acini per bees) [59] with the Saisam 5.0.1 software (Microvision®).

#### Abdomen gene expression

The abdomens of 10 bees per cage were homogenized in 1 ml of Trizol reagent (Invitrogen®) with a TissueLyser (Qiagen®) (4 x 30 s at 30 Hz). The mixture was incubated for 5 min at room temperature and after centrifugation (12,000 g for 30 s at 4° C) 500 µl of the supernatant was used for RNA extraction. One hundred µl of Chloroform (Sigma®) were added and the solution was incubated for 3 min and centrifuged (12,000 g for 15 min at 4° C). The aqueous phase was mixed with an equal volume of 70% ethanol (Sigma®) and transferred into a Qiagen RNeasy column. RNA extraction was carried out as indicated in the Qiagen RNeasy kit for total RNA with on-column DNase I treatment (Qiagen®). For cDNA synthesis, 1,000 ng of RNA per sample were reverse-transcribed using the High capacity RNA to cDNA Kit (Applied Biosystems). cDNA samples were diluted ten-fold in molecular grade water.

The expression level of *vitellogenin* and *transferrin* was determined by quantitative PCR using a StepOne-Plus Real-Time PCR Systems (Applied Biosystems®) and the SYBR green detection method including the ROX passive reference dye. Three μl cDNA were mixed to 5 μl SYBR Green PCR Master Mix (Applied Biosystems®), 1 μl of forward primer (10 µmol) and 1 μl of reverse primer (10 µmol) of candidate genes. Cycle threshold (Ct) values of selected genes were normalized to the housekeeping gene *Actin* using the comparative quantification method (delta Ct method). Primer sequences (5’–3’) were: *vitellogenin* forward: TTGACCAAGACAAGCGGAACT, reverse: AAGGTTCGAATTAACGATGAA [60]; *transferrin* gene: forward: AGCGGCATACTCCAGGGAC, reverse: CGTTGAGCCTGATCCATACGA [61]; *Actin* forward: TGCCAACACTGTCCTTTCTG, reverse: AGAATTGACCCACCAATCCA.

### Influence of pollen quality and diversity on bee tolerance to *Nosema* *ceranae*

For the experiment on bee tolerance to *Nosema* *ceranae* , groups of 70 one-day old bees were placed in cages and reared for 7 days with one of the pollen diet. For each pollen diet, one group was infected with *Nosema* and one group was *Nosema*-free, giving 12 treatment groups. On day 10, 28 bees per cage were flash frozen in liquid nitrogen and stored at -20° C until analysis of glutathione-S-transferase, alkaline phosphatase and phenoloxidase. The other 42 bees were used to determine the influence of pollen diet and *Nosema* *ceranae* on bee survival. Dead bees were counted daily and removed from the cages until half of the bees had died. The experiment was repeated 9 times per treatment group (pollen diet, *Nosema* infection).

#### Bee infection with *Nosema* *ceranae*

*Nosema* spores were isolated from infected colonies. Ten abdomens of forager bees were crushed in 2 μl of distilled water using an electric grinder (Ultra Turrax ® T18 basic, IKA®). Homogenates were then filtered with paper Whatman No. 4, and the filtrate was supplemented with 10 ml of distilled water. Solutions were centrifuged three times at 800 g for 6 minutes and each time the spore pellet was resuspended in 10 ml of distilled water. Species identification was performed as in Alaux et al. [62] and the spore concentration was determined using a haemocytometer. To equally infect bees with a *Nosema* *ceranae* *inoculum*, bees were fed individually with 2 µl of freshly prepared 50% sucrose solution containing 100,000 spores, which is known to cause an infection in worker bees [63–65]. Control bees were fed with a sucrose solution. At the end of the experiment, the guts of the bees were analyzed: no spores were found in the control bees but the infected bees were heavily parasitized (data not shown).

#### Enzyme analysis

Enzyme activities were assayed in different bee tissues: GST in the gut and head, ALP in the gut and PO in the abdomen devoid of gut. All analyses were performed on 3 pools of 3 bees per cage and in triplicate. Samples were homogenized at 4° C with TissueLyser (Qiagen®) (5 x10 s at 30 Hz) in the extraction buffer (10 mM NaCl, 1% (w/v) Triton X-100, 40 mM sodium phosphate pH 7.4, containing a mixture of 2 mg/ml of antipain, leupeptin and pepstatin A, 25 units/ml of aprotinin and 0.1 mg/ml of trypsin inhibitor) based on the weight of each pool (10% w/v extract). The homogenate was then centrifuged at 4° C for 20 min at 15,000 g. The enzymatic activities in supernatant were assayed in microplates with a BioTek Synergy HT100 spectrophotometer (BioTek Instruments®). GST was assayed in a reaction medium (200 µL final volume) containing 10 µl of tissue extract and 1 mM EDTA, 2.5 mM reduced glutathione, 1 mM 1-chloro-2,4-dinitrobenzene and 100 mM Na/K-phosphate pH 7.4. GST activity was followed spectrophotometrically at 340 nm by measuring the conjugation of 1-chloro-2,4-dinitrobenzene with reduced glutathione for 5 min at 25° C. ALP was assayed in a reaction medium (200 µL final volume) containing 10 µl of tissue extract and 20 mM of MgCl_2_, 2 mM of *p*-nitrophenyl phosphate as a substrate and 100 mM Tris-HCl pH 8.5 [66]. ALP activity was followed by measuring *p*-nitrophenyl phosphate hydrolysis at 410 nm for 5 min at 25° C. PO was assayed in a reaction medium (200 µL final volume) containing 50 µl of tissue extract and 200 mM NaCl, 0,4 mg/mL L-Dopa (3,4-Dihydroxy-L-phenylalanine), 100 mM sodium phosphate pH 7.2). PO activity was followed at 490 nm by measuring the conversion of L-Dopa to melanin for 10 min [62].

#### Statistical analysis

The statistical analysis was performed using the statistical software R [67]. Since the data were not normally distributed, the influence of pollen quality and diversity on hypopharyngeal gland development, *vitellogenin* and *transferrin* expressions, and enzymatic activities was analyzed using Kruskal-Wallis and Dunn’s multiple comparison tests. To analyze survival data obtained during the 50 days of experiment, we transformed the data in survival table and the remaining bees were considered alive at the day 50. Consequently, we used a Cox proportional hazards regression model, with R functions (coxph) and the package [survival] [68] to analyze the effect of *Nosema*, pollen and Nosema x pollen interaction on bee survival. Then, the effects of *Nosema* for each pollen diet and the effect of each pollen in non- and *Nosema*-parasitized bees on survival were tested. For non- and *Nosema*-parasitized bees, the influence of pollen diets on enzyme activities was determined using Kruskal-Wallis and Dunn’s multiple comparison tests. For each pollen diet, the effect of *Nosema* parasitism on enzymes activities was analyzed using Mann-Whitney U tests. Finally, in order to better understand the underlying mechanisms of bee longevity, we assessed the link between LT50 (day on which 50% of the bees had died in each cage based on the raw data) and enzyme activities (average value of the 3 analyzed pools per cage) using Spearman correlation for healthy and parasitized bees.

## Results

### Pollen diet nutritional factors

The nutritional value of each pollen was characterized before testing their effects on bees (Table 1). We did not detect the presence of pesticides in the four pollens that composed the different diets (Table S1). Contrary to lipids and sugars, the levels of proteins, amino acids and antioxidant capacity varied greatly between pollens. Therefore, pollen diets could be ranked according to their protein content as follows (from the poorest to the richest): *Cistus*, *Erica*, Mix (25% of each pollen), *Castanea* and *Rubus*. Exactly the same trend was found when looking at amino acids and antioxidants levels. The difference between *Cistus* and *Rubus* was especially striking with the latter having about twice as many proteins and amino acids, and almost five times greater antioxidant capacity. However, the lipid and sugar contents, which did not vary as much, followed different patterns. For example, *Erica* pollen was the richest in lipids but the poorest in sugars, and the other way round for *Rubus* pollen.

**Table 1:** Nutritional factor contents in the different pollen **diets**.

Pollen diets	Proteins (%)	Lipids (%)	Sugars (%)	Amino acids (g)	Antioxidants (µmol)
*Cistus*	12	6.9	5.2	11.9	103
*Erica*	14.8	7.4	4.8	16.27	196
*Castanea*	21.6	6.6	5.0	18.68	399
*Rubus*	22	6.4	6.7	19.98	475
Mix	17.6	6.8	5.4	16.71	293

Mix indicates the pollen diet composed of 25% of each monofloral pollen. Pollen proteins, lipids and sugars are expressed as percent of pollen dry matter. The antioxydant power is expressed in µmol of Trolox equivalent/g of pollen. The amino acids are expressed in g/100g of pollen.

All pollen diets contained the same amino acids including the 10 essential amino acids required for the bee adult development [69]: arginine, histidine, lysine, tryptophan, phenylalanine, methionine, threonine, leucine, isoleucine, and valine (Table S2). As for protein contents, most amino acids were in lower amounts in the *Cistus* pollen (notably the 10 essential amino acids) and in higher amounts in the *Rubus* pollen, whereas *Erica* and *Castanea* pollens had intermediary levels. Only proline was at the highest amount in *Cistus* pollen.

Regarding individual sugars, only glucose and fructose were found in all pollens (Table S3). Trehalose, a major hemolymph sugar of bees, was present in *Cistus* and *Castanea* pollens. Finally, erlose was only found in Castanea pollen, which contained all analyzed sugars.

### Influence of pollen quality and diversity on nurse bee physiology

**Figure 1:**
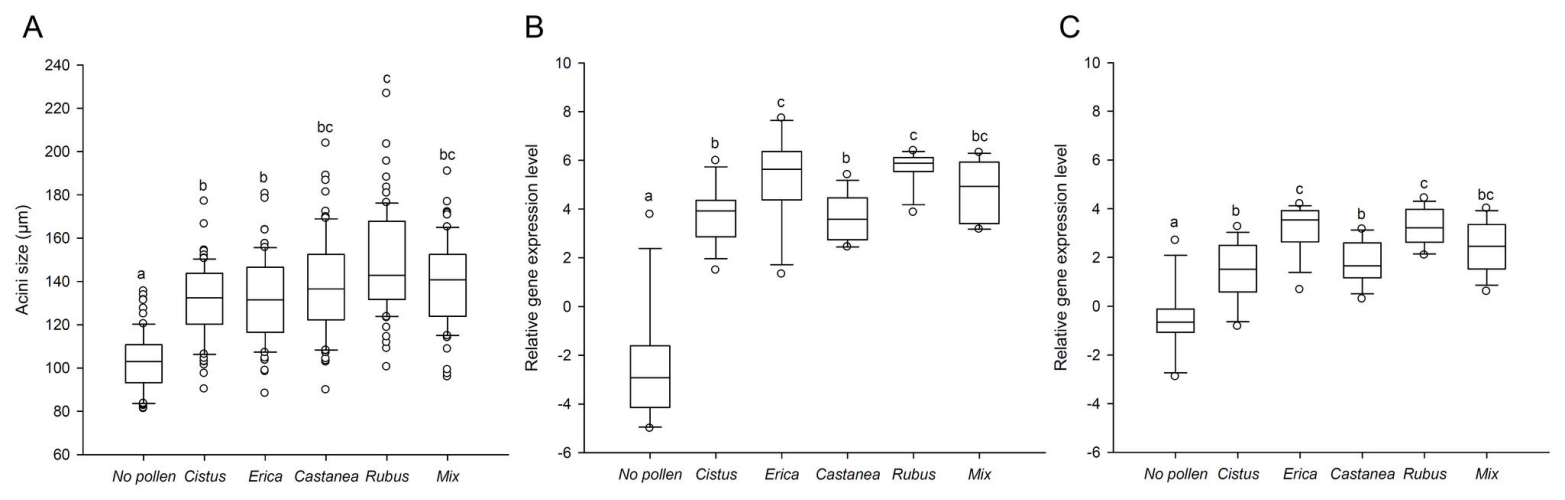
Effects of pollen quality and diversity on nurse physiology. (A) Size of hypopharyngeal gland acini, (B) *vitellogenin* and (C) *tansferrin* expression levels. Box plots are shown for 5 and 10 bees/replicate for the glands and each gene, respectively (*n* = 14 replicates giving 70 and 140 bees/pollen diet for the glands and each gene, respectively). Different letters indicate significant differences between pollen diets (*p* < 0.05, Kruskal-Wallis and Dunn’s multiple comparison tests). Boxes show 1st and 3rd interquartile range with line denoting median. Whiskers encompass 90% of the individuals, beyond which each outliers are represented by circles.

Pollen feeding modified the hypopharyngeal glands development (Kruskal-Wallis test, *H* = 143.84, *p* < 0.001; Figure 1A), which was reduced in bees reared without pollen but varied depending on pollen quality, since acini were more developed in bees fed with *Rubus* pollen compared to bees fed with *Cistus* and *Erica* pollen (Figure 1A). The gland development of bees fed with the polyfloral blend was not different from bees provided with the monofloral diets (139.5 ± 2.3 µm) but was almost equal to the average gland development induced by the four diets (137.5 ± 4.1 µm).

The expression level of *vitellogenin* and *transferrin* was significantly affected by the different pollen diets (*vitellogenin*: Kruskal-Wallis test, *H* = 43.13, *p* < 0.001, Figure 1B; *transferrin*: Kruskal-Wallis test, *H* = 42.31, *p* < 0.001, Figure 1C), with a higher expression in bees fed with pollen than in bees that did not receive pollen (Figure 1C). Interestingly, the quality of pollen diet also shaped the expression of both genes since *Erica* and *Rubus* pollen triggered the highest expression of *vitellogenin* and *transferrin* (Figure 1B and C). The influence of the polyfloral diet was not different from that of the others diets (*vitellogenin*: 4.8 ± 0.3 and *transferrin*: 2.4 ± 0.3), and corresponded to the average gene expression level induced by the four monofloral diets (*vitellogenin*: 4.6 ± 0.5 and *transferrin*: 2.4 ± 0.5).

### Influence of pollen quality and diversity on bee tolerance to *Nosema* *ceranae*

**Figure 2:**
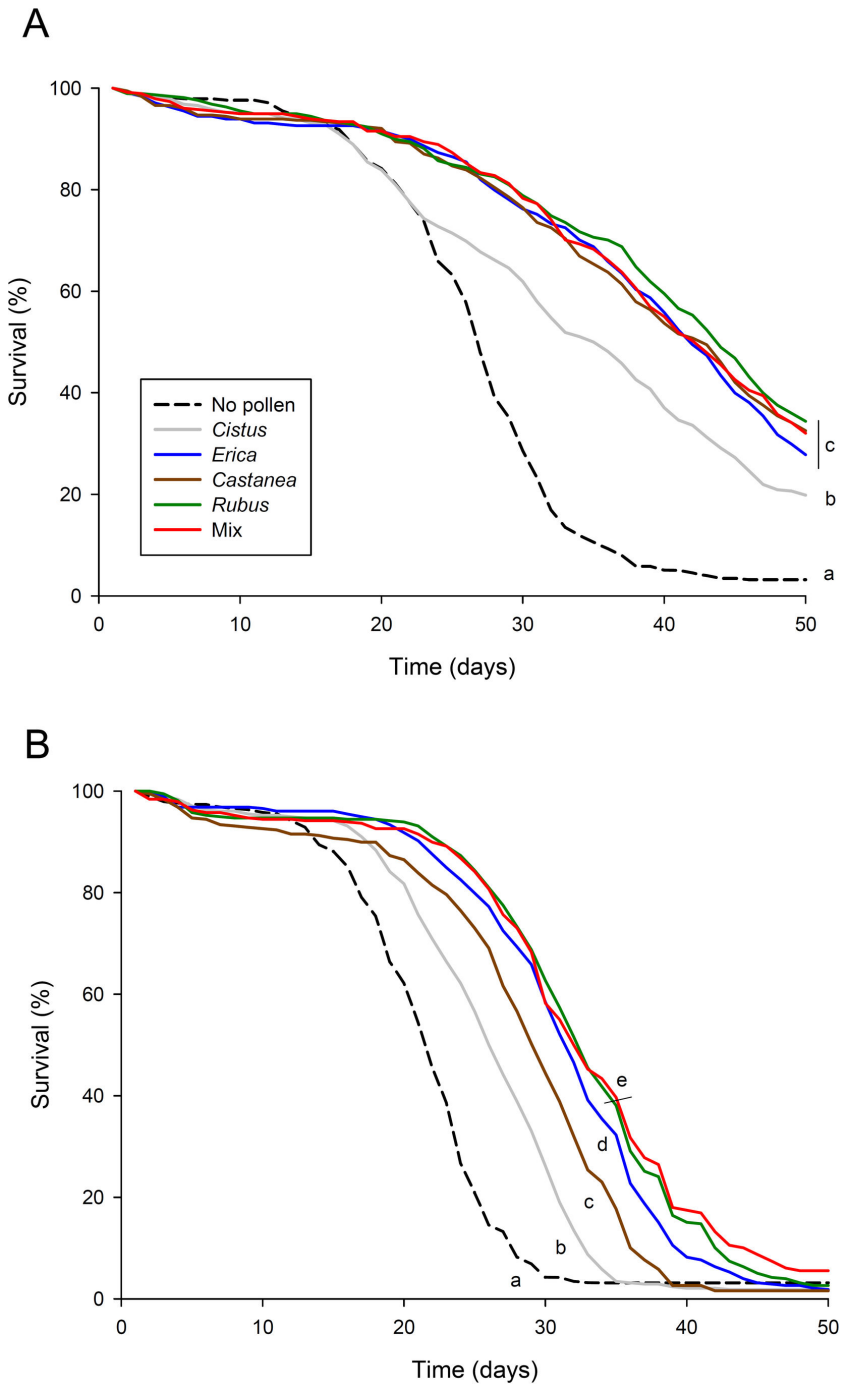
Effects of pollen diet and *Nosema ceranae* infection on bee survival. Data show the percentage of survival over 50 days for (A) non-parasitized and (B) *Nosema*-parasitized bees (9 replicates/pollen diet). Different letters denote significant differences between pollen diets in non-parasitized or *Nosema*-parasitized bees (*p* < 0.05, Cox proportional hazards regression model).

*Nosema* parasitism and pollen diets decreased and increased the survival of bees, respectively (Cox model, *p* < 0.001 for each factor, Figure 2). *Nosema* effect was observed regardless of the type of pollen diet (*p* < 0.001 for each pollen diet, Figure 2) and pollen diets modified the survival of bees regardless of the exposure to *Nosema* (Figure 2 and Table 2). However, we found a significant interaction between *Nosema* and pollen diets (*p* < 0.001, Figure 2). Except for the *Cistus* pollen, the quality and diversity of pollen diet did not influence the survival of healthy bees, but it mattered when bees were parasitized (Figure 2 and Table 2). Indeed, we observed a significant hierarchical influence of monofloral pollens on the survival of parasitized bees with the following order from the least to the most beneficial pollen: *Cistus* < *Castanea* < *Erica* < *Rubus*. In addition, bees fed with the polyfloral pollen blend lived significantly longer than bees provided with *Cistus*, *Erica* and *Castanea* pollen but there was no significant difference with bees provided with *Rubus* pollen (Figure 2 and Table 2).

**Table 2:** Comparative effects of pollen diets on the survival of (A) non- and (B) *Nosema*-parasitized bees.

	*Cistus*	*Erica*	*Castanea*	*Rubus*	Mix
**A**					
No pollen	< 0.0001	< 0.0001	< 0.0001	< 0.0001	< 0.0001
*Cistus*		< 0.0001	< 0.0001	< 0.0001	< 0.0001
*Erica*			0.47	0.1	0.35
*Castanea*				0.36	0.84
*Rubus*					0.47
**B**					
No pollen	< 0.0001	< 0.0001	< 0.0001	< 0.0001	< 0.0001
*Cistus*		< 0.0001	< 0.0001	< 0.0001	< 0.0001
*Erica*			< 0.0001	0.047	0.007
*Castanea*				< 0.0001	< 0.0001
*Rubus*					0.42

P-values from the Cox proportional hazards regression model are reported.

**Figure 3:**
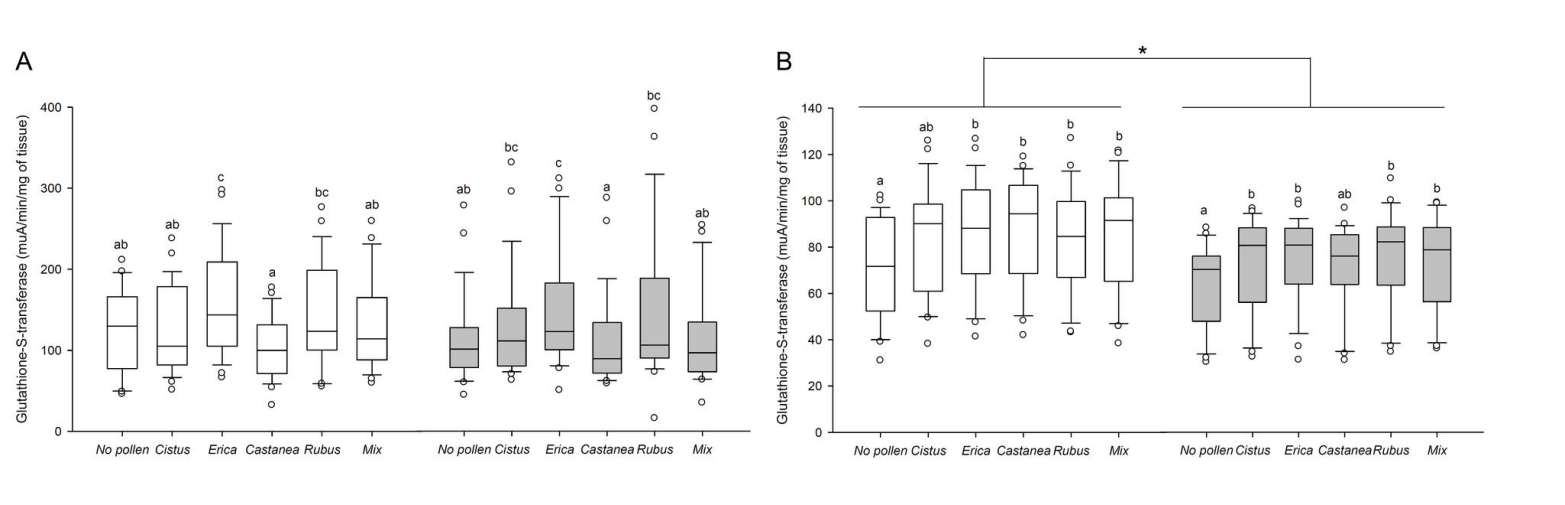
Effects of pollen diet and *Nosema ceranae* infection on glutathione S-transferase. The enzyme activity was assessed in (A) the guts and (B) the heads of bees. Box plots are shown for 3 pools of 3 bees/replicate (*n* = 9 replicates giving 81 bees total/pollen diet). Different letters denote significant differences between pollen diets in non-parasitized (white box plots) or *Nosema*-parasitized bees (grey box plots) (*p* < 0.05, Kruskal-Wallis and Dunn’s multiple comparison tests) and * indicate significant differences between parasitized and non-parasitized bees for each pollen diet (*p* < 0.05, Mann-Whitney U tests). Boxes show 1st and 3rd interquartile range with line denoting median. Whiskers encompass 90% of the individuals, beyond which each outliers are represented by circles.

When looking at the bee physiology, *Nosema* did not affect gut GST activity (Figure 3A). However, pollen diets did modify GST level in both healthy and parasitized bees (Kruskal-Wallis test, *H* = 35.73, *p* < 0.001 and Kruskal-Wallis test, *H* = 32.73, *p* < 0.001, respectively, Figure 3A) and the highest activity was observed with *Erica* pollen diet (Figure 3A). In the head, GST activity was significantly lower in bees infected with *Nosema* (Figure 3B) but was higher in bees fed with pollen regardless of exposure to *Nosema* (Kruskal-Wallis test, *H* = 22.06, *p* < 0.001 and Kruskal-Wallis test, *H* = 27.28, *p* < 0.001, respectively, Figure 3B). Contrary to what was observed in the gut, the type of pollen diet did not affect head GST level.

**Figure 4:**
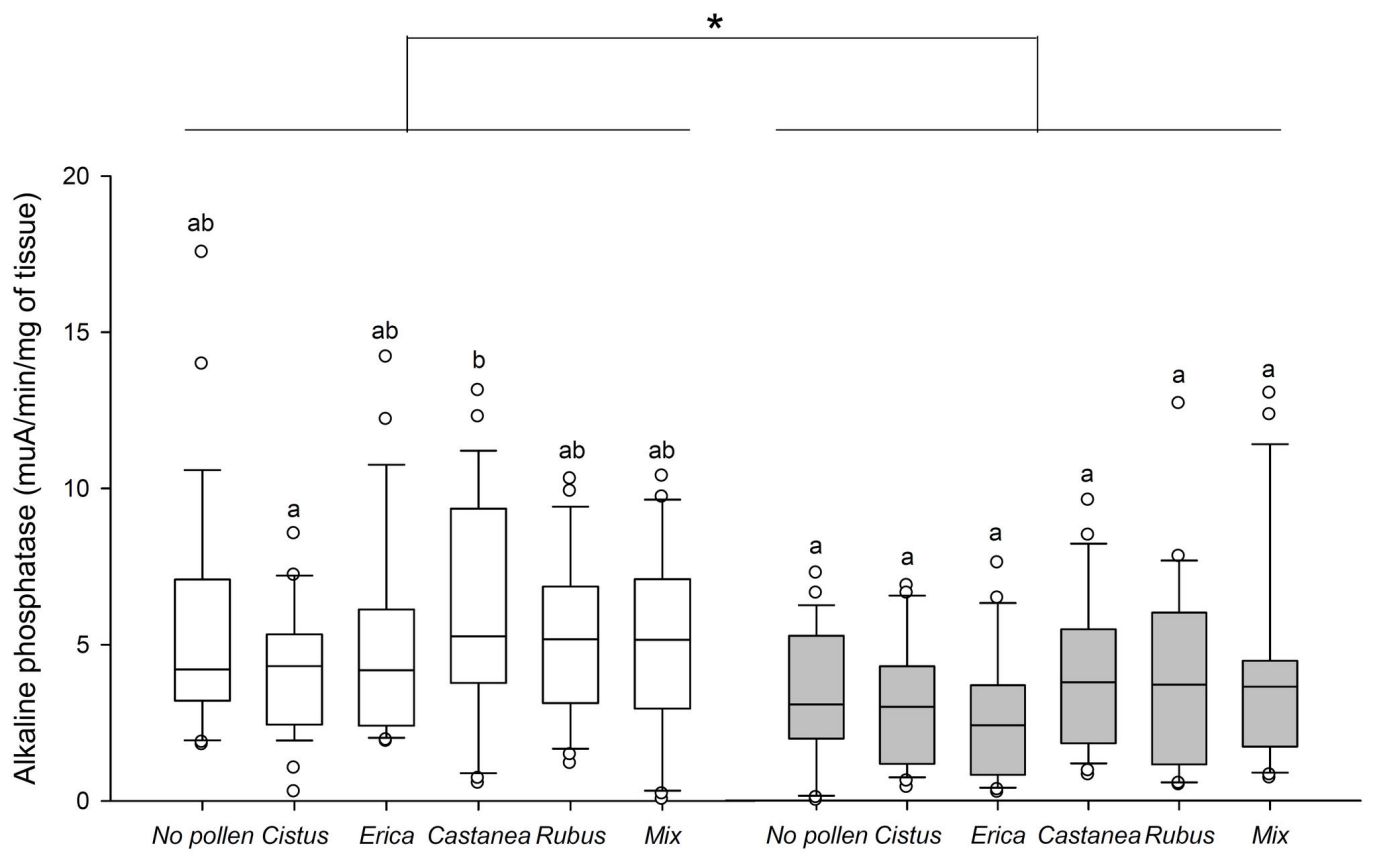
Effect of pollen diet and *Nosema ceranae* infection on gut alkaline phosphatase. Box plots are shown for 3 pools of 3 bees/replicate (*n* = 9 replicates giving 81 bees total/pollen diet). Different letters denote significant differences between pollen diets in non-parasitized (white box plots) or *Nosema*-parasitized bees (grey box plots) (*p* < 0.05, Kruskal-Wallis and Dunn’s multiple comparison tests) and * indicate significant differences between parasitized and non-parasitized bees for each pollen diet (*p* < 0.05, Mann-Whitney U tests). Boxes show 1st and 3rd interquartile range with line denoting median. Whiskers encompass 90% of the individuals, beyond which each outliers are represented by circles.

*Nosema* *ceranae* caused a decrease in ALP activity whatever the pollen diet (Figure 4). However, besides a higher activity induced by *Castanea* pollen compared to *Cistus* pollen in healthy bees, the quality and the diversity of pollen supply did not affect the ALP activity in the bee gut (healthy bees: Kruskal-Wallis test, *H* = 14.29, *p* = 0.013 and parasitized bees: Kruskal-Wallis test, *H* = 12.54, *p* = 0.028, Figure 4).

**Figure 5:**
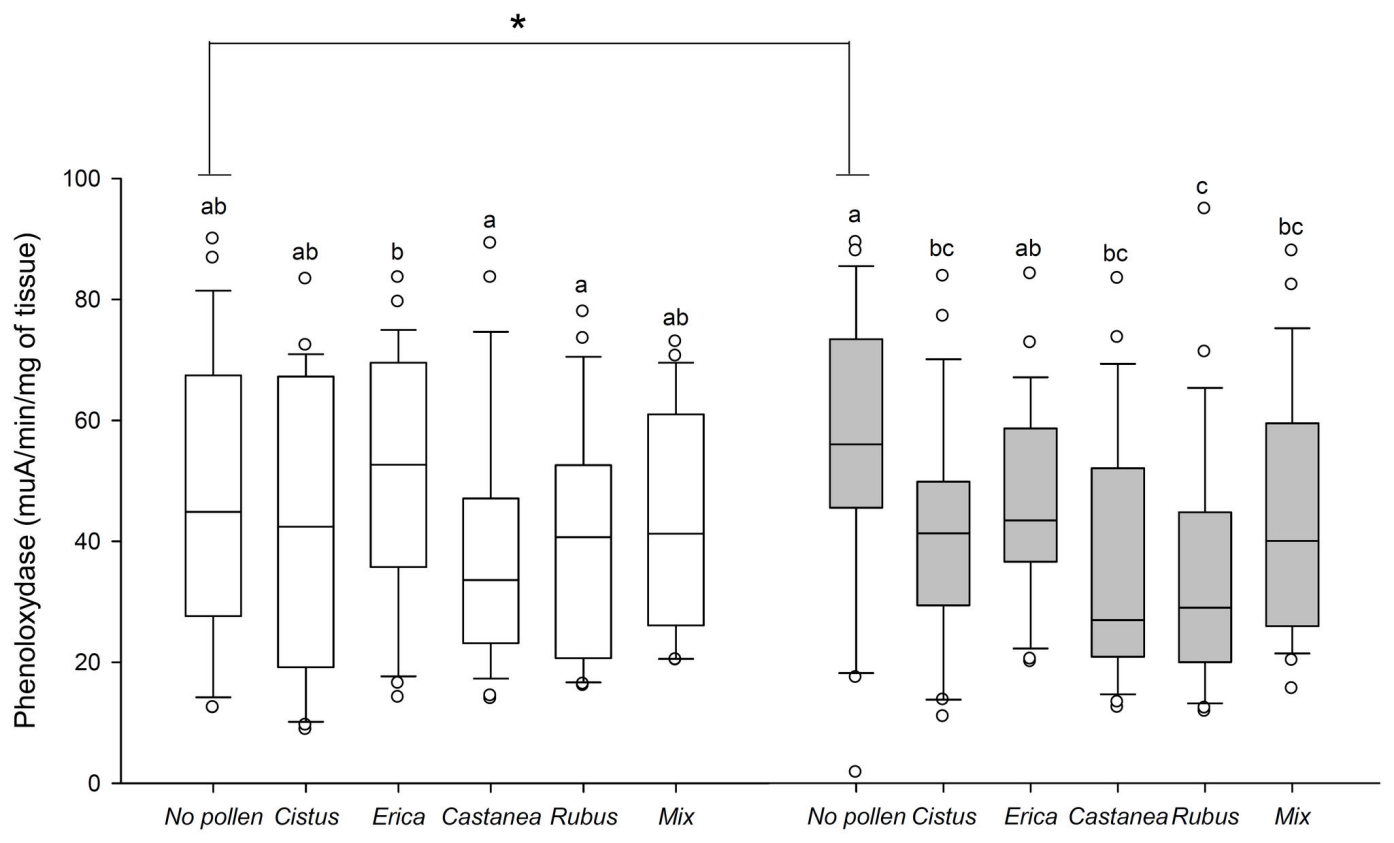
Effect of pollen diet and *Nosema ceranae* infection on phenoloxidase. Box plots are shown for 3 pools of 3 bees/replicate (*n* = 9 replicates giving 81 bees total/pollen diet). Different letters denote significant differences between pollen diets in non-parasitized (white box plots) or *Nosema*-parasitized bees (grey box plots) (*p* < 0.05, Kruskal-Wallis and Dunn’s multiple comparison tests) and * indicate significant differences between parasitized and non-parasitized bees for each pollen diet (*p* < 0.05, Mann-Whitney U tests). Boxes show 1st and 3rd interquartile range with line denoting median. Whiskers encompass 90% of the individuals, beyond which each outliers are represented by circles.

*Nosema* *ceranae* induced a significant increase of PO activity in bees deprived of pollen (Figure 5). In infected bees the immune enzyme activity was lower in the presence of pollen, except for *Erica* (Kruskal-Wallis test, *H* = 49.64, *p* < 0.001, Figure 5). In healthy bees, pollen intake had limited effect on PO activity (Kruskal-Wallis test, *H* = 19.24, *p* < 0.001, Figure 5). Only *Erica* pollen elicited a significant higher activity when compared to *Castanea* and *Rubus* pollen.

**Figure 6:**
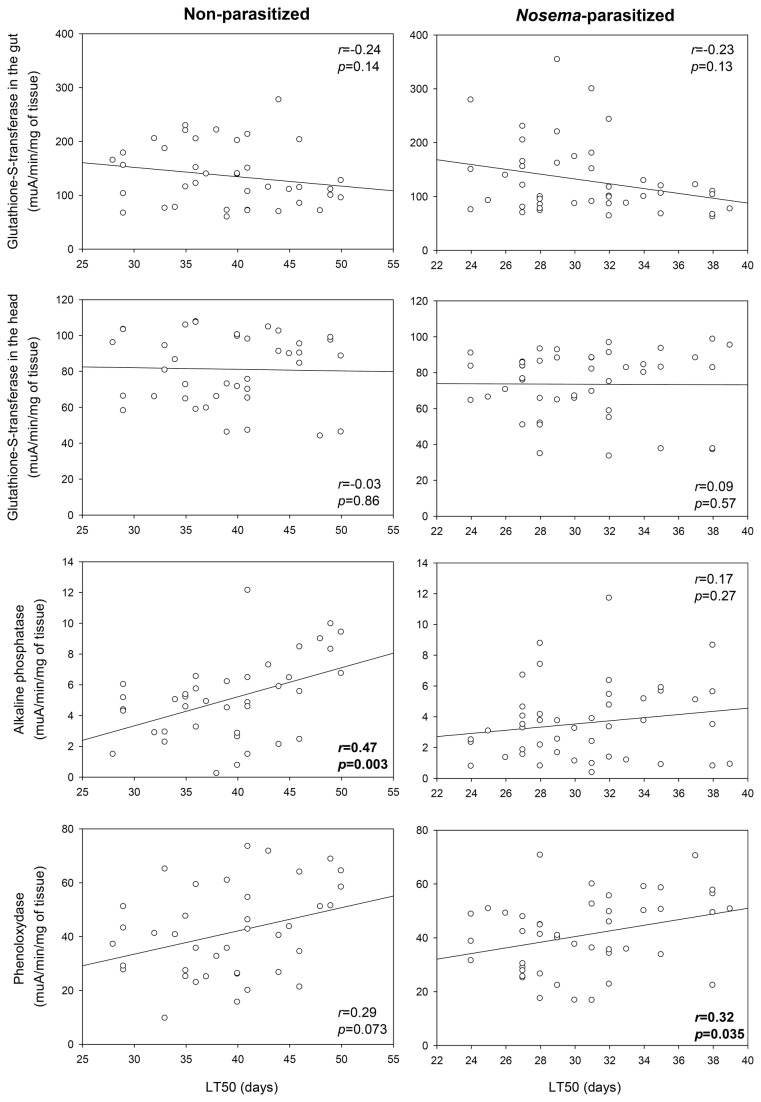
Correlations between LT50 and enzyme activities in non- and *Nosema*-parasitized bees. *r* and *p*-values are shown. LT50: day on which 50% of the bees had died in each cage.

Lastly, we determined whether the LT50 of bees was linked to the activity of the different investigated enzymes. In healthy bees longevity was positively correlated with ALP activity (i.e., ALP activity explained 50% of bee longevity), but when bees were *Nosema*-infected, longevity was positively linked to PO activity (Figure 6).

## Discussion

The results of this study support the idea that the nutritional quality and diversity of pollen nutrition can shape bee health. Indeed, we found that both bee physiology and tolerance to a parasite varied depending on the type of pollen diet, suggesting that not only does the availability but also the quality of environmental resources matter.

The type of pollen provided to the bees had significant effects on the nurse bee physiology. Bees fed with the protein-richest pollen (*Rubus*) presented the most developed acini and the highest expression level of *vitellogenin* and *transferrin*. This tends to confirm previous studies that showed that the hypopharyngeal gland development is linked to the level of proteins in the diet [29,31]. However, the other pollen diets did not significantly induce different gland developments, which could be explained by a too small range of protein and/or other nutritional factors contents. Pollen feeding also increased the expressions of *vitellogenin* and *transferrin*. Since both genes are expressed in the fat bodies, the main site of nutrient storage, and pollen promotes the development of fat bodies [19], it is reasonable to expect an increase in both gene expression levels, as previously found for *vitellogenin* after consumption of proteins [70]. However, the expressions of *vitellogenin* and *transferrin* in bees fed with *Erica* pollen were not different from bees fed with *Rubus* pollen, although *Erica* had a lower amount of proteins. This suggests that their expression is not only sensitive to the protein level but also to other nutritional factors. When looking at the nutritional factors, we found that *Erica* pollen had the highest content in lipids, which might have promoted the increase of fat bodies and therefore the expression of both genes, since fat body tissues are also the primary site of lipid metabolism (e.g. fatty acid synthesis and triacylglyceride production) [71]. This potential role of lipids in *vitellogenin* synthesis would further confirm that they are essential to the nurse physiology [72] and the production of brood [73]. In addition, it is interesting to note that *vitellogenin* and *transferrin* had similar expression patterns according to the different pollen diets. This covariation in gene expression was also found in previous works studying the potential role of those genes in ovary development [38,39].

The quality of pollen also influenced the tolerance of bees to a parasite ( *Nosema* *ceranae* ). As expected, infection by *Nosema* decreased the survival of bees [49,64] and pollen nutrition increased the survival of both healthy and parasitized bees. Except for bees fed with the protein-poorest pollen (*Cistus*), we did not observe a difference in survival between the different pollen diets when bees where non-parasitized. However, pollen quality had a strong influence when bees were parasitized by the microsporidia; the survival of bees was significantly different between the four different monofloral diets (from the least to the most beneficial pollen: *Cistus* < *Castanea* < *Erica* < *Rubus*). This suggests that the quality of pollen nutrients might have no or limited consequences on the physiology of bees when they are healthy, but it might affect their capacity to tolerate an external stress like parasites. The positive influence of *Rubus* pollen as compared to *Cistus* pollen has also been proved when looking at the effect of diet quality on larvae weight in bumble bees [74]. The extremely high protein and antioxidant levels of *Rubus* pollen, as compared to *Cistus* pollen, could explain the greater survival of infected bees fed with the former pollen. Notably proteins are known to improve bee survival (see 4 for a review). High levels of amino acids could play an important role too, since ten of them are essential to the bees in specific concentrations [69]. However, the hierarchical influence of monofloral diet was not linked to the protein, amino acid or antioxidant levels, e.g. bees fed with *Erica* pollen (14.8% of protein) lived longer than bees fed with *Castanea* (21.6% of protein). *Erica* pollen had actually the highest lipid content and promoted a higher production of *vitellogenin* than *Castanea* pollen (Figure 1B). The positive influence of *vitellogenin* on bee lifespan [36] might then contribute to the increased survival of parasitized bees supplied with *Erica* pollen. This suggests that the quality of pollen should not be estimated based on a single or few nutritional factors, but by taking all the nutritional factors as a whole.

Regarding the defense mechanism, the general activity of GST (detoxification), ALP and PO (immunity) changed too according to the pollen diets, but we did not observe a pattern similar to the bee survival. Therefore it was not possible to link the influence of diet quality on bee survival to the activity level of those enzymes. Moreover, the patterns of enzymes activity were not modified by *Nosema* infection, but the general level of head GST and ALP was reduced, confirming a previous study [49]. However, an increase of GST activity in the gut of *Nosema*-parasitized bees has been previously reported [48,49], likely to protect the host from the oxidative stress induced by the parasite [47]. The lack of GST response in our study could be due to the diet, since we did not use a commercial mixture of proteins, amino acids and vitamins as in both previous studies, which could have promoted a GST response. Interestingly, the activity profile of GST in the gut was very similar to the expression profile of *vitellogenin* and *transferrin* according to the different diets, with *Erica* and *Rubus* pollen giving the highest activity. However, nothing is known about the relationship between GST and those two genes. Regarding PO activity, it is well-known in other insects that PO level can be influenced by the diet quality [75–77]. Indeed, melanogenesis, regulated by PO through the synthesis of melanin (a nitrogen-rich quinone polymer), might be costly in nitrogen [78] and thus sensitive to variations in nitrogen resources. However, it did not vary between pollen diets in a previous study [19] and, in our study, it was only higher with *Erica* pollen. Therefore, further investigations are needed to better understand the relationship between pollen diet and PO activity in bees.

Pollen dietary diversity was not associated with an improvement of nurse physiology, as reflected by the measured physiological parameters. The influence of the polyfloral diet actually came down to the average of each monofloral pollen influence. This suggests that a high-quality monofloral pollen may be better than a mixture of lower nutritional quality as found for brood rearing [79,80]. However, it is likely that different physiological factors in bees are not affected equally by the pollen diet. This has been observed in a recent study showing a higher activity of glucose oxidase in bees fed with a polyfloral pollen blend as compared to monofloral pollen, but PO activity and hemocyte count were not affected by the polyfloral diet [19]. This is further confirmed by our study, since the polyfloral blend had a positive influence on the survival of parasitized bees. It did not correspond to the average of each pollen effect, but was higher than *Cistus,* *Castanea* and *Erica* pollens and to the same level than *Rubus* pollen. This trend was not observed in healthy bees suggesting again that nutritional quality can significantly affect the susceptibility of individuals to parasites. It is not known whether the increase in the survival of bees fed with the polyfloral blend was due to the combination of the four pollens or the simple presence of *Rubus* pollen, although it contained a quarter of this pollen. Similar results were found by Foley et al. [81], who observed a decreased susceptibility to the fungal parasite *Aspergillus* of bee larvae fed with a specific pollen or with a mixture.

Finally, in order to decipher some of the underlying physiological mechanisms involved in bee health, we determined whether the activity of GST, PO and ALP were associated to an increase of survival in healthy or parasitized bees. Survival was positively associated to ALP and PO activity in healthy and *Nosema*-infected bees, respectively. In mammals, ALP is involved in the regulation of nutrient absorption (notably lipids), detoxification of bacterial lipopolysaccharide, intestinal tolerance to commensal bacteria, prevents bacterial invasion and reduces intestinal inflammation, playing thus a pivotal role in intestine health (see 51 for a review). It is not known whether ALP has similar roles in insects but there are structural and functional homologies between insect and mammal ALPs [82]. In addition, the correlation between ALP activity and bee survival suggests that this enzyme might be important in insect health. When its activity was decreased by *Nosema* infection, it was no longer linked to bee survival. In that case, the survival rate was associated to PO activity. However, except in the absence of pollen, parasitized bees did not mount a PO immune response, which supports the idea that the bee survival was simply linked to a higher basal activity of PO.

In conclusion, pollens are not equal regarding their effects on bee health and a polyfloral blend is not necessarily better than a monofloral pollen of good nutritional values (e.g. *Rubus* pollen). However, when bees are infected (by *N* *. ceranae* here), the availability of different floral resources can cover the limited influence of some pollens and improve the tolerance to the infection to the level of a rich pollen. Pollinating areas of bees are currently changing due to intensification of agriculture and landscape alteration, and bees are often confronted with decreasing availability and diversity of resources in time and space. Global climate change is also expected to modify the environmental resources of bees due to changes in plant phenology and distribution [83]. Therefore, maintaining and/or developing floral resources within agro-ecosystems is needed to prevent the negative impact of human activity and sustain the bee population [7].

## Supporting Information

Table S1 **List of pesticides analyzed in the pollen diets.**
(XLSX)

Table S2 **Amino acids present in the different pollens.** Their concentration is expressed in g/100 g of pollen.
(DOCX)

Table S3 **Sugars present in the different pollens.** Their quantity is expressed in mg per g of pollen. *nd*: not detected and *nq*: present but not quantifiable.
(DOCX)

## References

[1] Klein AM, Vaissière BE, Cane JH, Steffan-Dewenter I, Cunningham SA et al. (2007) Importance of pollinators in changing landscapes for world crops. Proc R Soc Lond B 274: 303-313. doi:10.1098/rspb.2006.3721. PubMed: 17164193. 10.1098/rspb.2006.3721 PMC1702377 17164193

[2] Gallai N, Salles JM, Settele J, Vaissiere BE (2009) Economic valuation of the vulnerability of world agriculture confronted with pollinator decline. Ecol Econ 68: 810-821. doi:10.1016/j.ecolecon.2008.06.014.

[3] Morse RA (1991) Honeybees forever. Trends Ecol Evol 6: 337-338. doi:10.1016/0169-5347(91)90043-W. PubMed: 21232501. 21232501 10.1016/0169-5347(91)90043-W

[4] Brodschneider R, Crailsheim K (2010) Nutrition and health in honey bees. Apidologie 41: 278-294. doi:10.1051/apido/2010012.

[5] Keller I, Fluri P, Imdorf A (2005) Pollen nutrition and colony development in honey bees, Part II. Bee World 86: 27-34.

[6] Haydak MH (1970) Honey bee nutrition. Annu Rev Entomol 15: 143-156. doi:10.1146/annurev.en.15.010170.001043.

[7] Decourtye A, Mader E, Desneux N (2010) Landscape enhancement of floral resources for honey bees in agro-ecosystems. Apidologie 41: 264-277. doi:10.1051/apido/2010024.

[8] Naug D (2009) Nutritional stress due to habitat loss may explain recent honeybee colony collapses. Biol Conserv 142: 2369-2372. doi:10.1016/j.biocon.2009.04.007.

[9] Van Engelsdorp D, Hayes J Jr., Underwood RM, Pettis J (2008) A survey of honey bee colony losses in the U.S., fall 2007 to spring 2008. PLOS ONE 3: e4071. doi:10.1371/journal.pone.0004071. PubMed: 19115015. 19115015 10.1371/journal.pone.0004071 PMC2606032

[10] Neumann P, Carreck NL (2010) Honey bee colony losses. J Apicult Res 49: 1-6. doi:10.3896/IBRA.1.49.1.01.

[11] Van Engelsdorp D, Meixner MD (2010) A historical review of managed honey bee populations in Europe and the United States and the factors that may affect them. J Invertebr Pathol 103: S80-S95. doi:10.1016/j.jip.2009.06.011. PubMed: 19909973. 19909973 10.1016/j.jip.2009.06.011

[12] Roulston TH, Buchmann SL (2000) A phylogenetic reconsideration of the pollen starch-pollination correlation. Evol Ecol Res 2: 627-643.

[13] Stanley RG, Linskens HF (1974) Pollen: Biology, biochemistry, management. Heidelberg, Germany: Springer Verlag.

[14] Crailsheim K, Schneider LHW, Hrassnigg N, Bühlmann G, Brosch U et al. (1992) Pollen consumption and utilization in worker honeybees (Apis mellifera carnica): dependence on individual age and function. J Insect Physiol 38: 409-419. doi:10.1016/0022-1910(92)90117-V.

[15] Crailsheim K (1992) The Flow of Jelly within a Honeybee Colony J Comp Physiol B 162: 681-689. doi:10.1007/BF00301617.

[16] Le Conte Y, Brunet J-L, McDonnell C, Dussaubat C, Alaux C (2011) Interactions between risk factors in honey bees. In: Sammataro D Yoder J Recent Investigations into the Problems with our Honey Bee Pollinators. Taylor & Francis Inc. pp. 215-222.

[17] Alaux C, Dantec C, Parrinello H, Le Conte Y (2011) Nutrigenomics in honey bees: digital gene expression analysis of pollen’s nutritive effects on healthy and varroa-parasitized bees. BMC Genomics 12: 496. doi:10.1186/1471-2164-12-496. PubMed: 21985689. 21985689 10.1186/1471-2164-12-496 PMC3209670

[18] Ament SA, Chan QW, Wheeler MM, Nixon SE, Johnson SP et al. (2011) Mechanisms of stable lipid loss in a social insect. J Exp Biol 214: 3808-3821. doi:10.1242/jeb.060244. PubMed: 22031746. 22031746 10.1242/jeb.060244 PMC3202514

[19] Alaux C, Ducloz F, Crauser D, Le Conte Y (2010) Diet effects on honeybee immunocompetence. Biol Lett 6: 562-565. doi:10.1098/rsbl.2009.0986. PubMed: 20089536. 20089536 10.1098/rsbl.2009.0986 PMC2936196

[20] Rinderer TE, Rothenbuhler WC, Gochnauer TA (1974) The influence of pollen on the susceptibility of honey-bee larvae to *Bacillus* larvae. J Invertebr Pathol 23: 347-350. doi:10.1016/0022-2011(74)90100-1. PubMed: 4833177. 4833177 10.1016/0022-2011(74)90100-1

[21] Degrandi-Hoffman G, Chen Y, Huang E, Huang MH (2010) The effect of diet on protein concentration, hypopharyngeal gland development and virus load in worker honey bees (*Apis mellifera* L.). J Insect Physiol 56: 1184-1191. doi:10.1016/j.jinsphys.2010.03.017. PubMed: 20346950. 20346950 10.1016/j.jinsphys.2010.03.017

[22] Rinderer TE, Elliott KD (1977) Worker honey bee response to infection with *Nosema apis* . J Econ Entomol 70: 431-433.

[23] Wahl O, Ulm K (1983) Influence of pollen feeding and physiological condition on pesticide sensitivity of the honey bee *Apis mellifera carnica* . Oecologia 59: 106-128. doi:10.1007/BF00388082. 25024157 10.1007/BF00388082

[24] Roulston TH, Cane JH (2000) Pollen nutritional content and digestibility for animals. Plant Syst Evol 222: 187-209. doi:10.1007/BF00984102.

[25] Herbert EW Jr., Shimanuki H (1978) Chemical composition and nutritive value of bee-collected and bee-stored pollen. Apidologie 9: 33-40. doi:10.1051/apido:19780103.

[26] Odoux JF, Feuillet D, Aupinel P, Loublier Y, Tasei JN et al. (2012) Territorial biodiversity and consequences on physico-chemical characteristics of pollen collected by honey bee colonies. Apidologie 43: 561-575. doi:10.1007/s13592-012-0125-1.

[27] Schmidt JO, Thoenes SC, Levin MD (1987) Survival of honey bees, *Apis mellifera* (Hymenoptera: Apidae), fed various pollen sources. J Econ Entomol 80: 176-183.

[28] Schmidt LS, Schmidt JO, Rao H, Wang W, Xu L (1995) Feeding preference and survival of young worker honey bees (Hymenoptera: Apidae) fed rape, sesame, and sunflower pollen. J Econ Entomol 88: 1591-1595.

[29] Standifer LN (1967) A comparison of the protein quality of pollens for growth stimulation of the hypopharyngeal glands and longevity of honey bees, *Apis mellifera* L. (Hymenoptera: Apidae). Insects Soc 14: 415-426. doi:10.1007/BF02223687.

[30] Maurizio A (1950) The influence of pollen feeding and brood rearing on the length of life and physiological condition of the honey bee. Bee World 31: 9-12.

[31] Pernal SF, Currie RW (2000) Pollen quality of fresh and 1-year-old single pollen diets for worker honey bees (*Apis mellifera* L.). Apidologie 31: 387-409. doi:10.1051/apido:2000130.

[32] Amdam GV, Page RE (2010) The developmental genetics and physiology of honeybee societies. Anim Behav 79: 973-980. doi:10.1016/j.anbehav.2010.02.007. PubMed: 20514137. 20514137 10.1016/j.anbehav.2010.02.007 PMC2875690

[33] Ament SA, Wang Y, Robinson GE (2010) Nutritional regulation of division of labor in honey bees: toward a systems biology perspective. Wiley Interdiscip. Rev Syst Biol Med 2: 566-576. 10.1002/wsbm.73 20836048

[34] Amdam GV, Norberg K, Fondrk MK, Page RE Jr. (2004) Reproductive ground plan may mediate colony-level selection effects on individual foraging behavior in honey bees. Proc Natl Acad Sci U S A 101: 11350-11355. doi:10.1073/pnas.0403073101. PubMed: 15277665. 15277665 10.1073/pnas.0403073101 PMC509206

[35] Amdam GV, Norberg K, Hagen A, Omholt SW (2003) Social exploitation of vitellogenin. Proc Natl Acad Sci U S A 100: 1799-1802. doi:10.1073/pnas.0333979100. PubMed: 12566563. 12566563 10.1073/pnas.0333979100 PMC149913

[36] Seehuus SC, Norberg K, Gimsa U, Krekling T, Amdam GV (2006) Reproductive protein protects functionally sterile honey bee workers from oxidative stress. Proc Natl Acad Sci U S A 103: 962-967. doi:10.1073/pnas.0502681103. PubMed: 16418279. 16418279 10.1073/pnas.0502681103 PMC1347965

[37] Amdam GV, Simões ZLP, Hagen A, Norberg K, Schrøder K et al. (2004) Hormonal control of the yolk precursor vitellogenin regulates immune function and longevity in honeybees. Exp Gerontol 39: 767-773. doi:10.1016/j.exger.2004.02.010. PubMed: 15130671. 15130671 10.1016/j.exger.2004.02.010

[38] Koywiwattrakul P, Sittipraneed S (2009) Expression of vitellogenin and transferrin in activated ovaries of worker honey bees, *Apis mellifera* . Biochem Genet 47: 19-26. doi:10.1007/s10528-008-9202-6. PubMed: 19096928. 19096928 10.1007/s10528-008-9202-6

[39] Koywiwattrakul P, Thompson GJ, Sitthipraneed S, Oldroyd BP, Maleszka R (2005) Effects of carbon dioxide narcosis on ovary activation and gene expression in worker honey bees, *Apis mellifera* . J Insect Sci 5: 36 PubMed: 17119618. 17119618 10.1093/jis/5.1.36 PMC1615243

[40] Nino EL, Tarpy DR, Grozinger CM (2013) Differential effects of insemination volume and substance on reproductive changes in honey bee queens (*Apis mellifera* L.). Insect Mol Biol. 10.1111/imb.12016 23414204

[41] Kucharski R, Maleszka R (2003) Transcriptional profiling reveals multifunctional roles for transferrin in the honeybee, *Apis mellifera* . J Insect Sci 3: 27 PubMed: 15841243. 15841243 10.1093/jis/3.1.27 PMC524666

[42] Higes M, Meana A, Bartolomé C, Botías C, Martín-Hernández R (2013) *Nosema ceranae* (Microsporidia), a controversial 21st century honey bee pathogen. Environ Microbiol Rep 5: 17-29. doi:10.1111/1758-2229.12024. PubMed: 23757127. 23757127 10.1111/1758-2229.12024

[43] Fries I (2010) *Nosema ceranae* in European honey bees (*Apis mellifera*). J Invertebr Pathol 103: S73-S79. doi:10.1016/j.jip.2009.06.017. PubMed: 19909977. 19909977 10.1016/j.jip.2009.06.017

[44] Higes M, Martin-Hernandez R, Meana A (2010) *Nosema ceranae* in Europe: an emergent type C nosemosis. Apidologie 41: 375-392. doi:10.1051/apido/2010019.

[45] Paxton RJ (2010) Does infection by *Nosema ceranae* cause "Colony Collapse Disorder" in honey bees (*Apis mellifera*)? J Apicult Res 49: 80-84. doi:10.3896/IBRA.1.49.1.11.

[46] Sies H (1997) Oxidative stress: oxidants and antioxidants. Exp Physiol 82: 291-295. PubMed: 9129943. 9129943 10.1113/expphysiol.1997.sp004024

[47] Buchon N, Broderick NA, Poidevin M, Pradervand S, Lemaitre B (2009) Drosophila intestinal response to bacterial infection: activation of host defense and stem cell proliferation. Cell Host Microbe 5: 200-211. doi:10.1016/j.chom.2009.01.003. PubMed: 19218090. 19218090 10.1016/j.chom.2009.01.003

[48] Vidau C, Diogon M, Aufauvre J, Fontbonne R, Viguès B et al. (2011) Exposure to sublethal doses of fipronil and thiacloprid highly increases mortality of honeybees previously infected by *Nosema ceranae* . PLOS ONE 6: e21550. doi:10.1371/journal.pone.0021550. PubMed: 21738706. 21738706 10.1371/journal.pone.0021550 PMC3125288

[49] Dussaubat C, Brunet JL, Higes M, Colbourne JK, Lopez J et al. (2012) Gut Pathology and Responses to the Microsporidium Nosema *ceranae* in the Honey Bee *Apis mellifera* . PLOS ONE 7: e37017. doi:10.1371/journal.pone.0037017. PubMed: 22623972. 22623972 10.1371/journal.pone.0037017 PMC3356400

[50] González-Santoyo I, Córdoba-Aguilar A (2012) Phenoloxidase: a key component of the insect immune system. Entomol Exp Appl 142: 1-16. doi:10.1111/j.1570-7458.2011.01187.x.

[51] Lallès JP (2010) Intestinal alkaline phosphatase: multiple biological roles in maintenance of intestinal homeostasis and modulation by diet. Nutr Rev 68: 323-332. doi:10.1111/j.1753-4887.2010.00292.x. PubMed: 20536777. 20536777 10.1111/j.1753-4887.2010.00292.x

[52] ISO 5983 (1997) Animal feeding stuffs. Determination of nitrogen content and calculation of crude protein content—Kjeldahl method. Switzerland, Geneva: International Organization for Standardization.

[53] Folch J, Lees M, Sloane Stanley GH (1957) A simple method for the isolation and purification of total lipides from animal tissues. J Biol Chem 226: 497-509. PubMed: 13428781. 13428781

[54] Louveaux J (1959) Recherches sur la récolte du pollen par les abeilles (*Apis mellifera* L.). Annales De L’abeille 2: 99.

[55] Commission regulation (EC) No 152/2009 of 27 January 2009 laying down the methods of sampling and analysis for the official control of feed. Official Journal Eur Union L 54 (26/2/2009): 23-37.

[56] Ou B, Hampsch-Woodill M, Prior RL (2001) Development and validation of an improved oxygen radical absorbance capacity assay using fluorescein as the fluorescent probe. J Agric Food Chem 49: 4619-4626. doi:10.1021/jf010586o. PubMed: 11599998. 11599998 10.1021/jf010586o

[57] Baude M, Leloup J, Suchail S, Allard B, Benest D et al. (2011) Litter inputs and plant interactions affect nectar sugar content. J Ecol 99: 828-837. doi:10.1111/j.1365-2745.2011.01793.x.

[58] AFNOR 15662 (2009) Foods of plant origin: Method versatile determination of pesticide residues by GC-MS and SL / SM / MS extraction / partition with acetonitrile and cleaned by SPE dispersed

[59] Crailsheim K, Stolberg E (1989) Influence of Diet, Age and Colony Condition Upon Intestinal Proteolytic Activity and Size of the Hypopharyngeal Glands in the Honeybee (*Apis-Mellifera L*). J Insect Physiol 35: 595-602. doi:10.1016/0022-1910(89)90121-2.

[60] Fischer P, Grozinger CM (2008) Pheromonal regulation of starvation resistance in honey bee workers (*Apis mellifera*). Naturwissenschaften 95: 723-729. doi:10.1007/s00114-008-0378-8. PubMed: 18414825. 18414825 10.1007/s00114-008-0378-8

[61] Thompson GJ, Yockey H, Lim J, Oldroyd BP (2007) Experimental manipulation of ovary activation and gene expression in honey bee (*Apis mellifera*) queens and workers: Testing hypotheses of reproductive regulation. J Exp Zool Aecol Genet Physiology 307A: 600-610. doi:10.1002/jez.415. PubMed: 17786975. 10.1002/jez.415 17786975

[62] Alaux C, Brunet JL, Dussaubat C, Mondet F, Tchamitchan S et al. (2010) Interactions between *Nosema* microspores and a neonicotinoid weaken honeybees (*Apis mellifera*). Environ Microbiol 12: 774-782. doi:10.1111/j.1462-2920.2009.02123.x. PubMed: 20050872. 20050872 10.1111/j.1462-2920.2009.02123.x PMC2847190

[63] Malone LA, Gatehouse HS (1998) Effects of *Nosema apis* infection on honey bee (*Apis mellifera*) digestive proteolytic enzyme activity. J Invertebr Pathol 71: 169-174. doi:10.1006/jipa.1997.4715.

[64] Higes M, García-Palencia P, Martín-Hernández R, Meana A (2007) Experimental infection of *Apis mellifera* honeybees with *Nosema ceranae* (Microsporidia). J Invertebr Pathol 94: 211-217. doi:10.1016/j.jip.2006.11.001. PubMed: 17217954. 17217954 10.1016/j.jip.2006.11.001

[65] Forsgren E, Fries I (2010) Comparative virulence of *Nosema ceranae* and *Nosema apis* in individual European honey bees. Vet Parasitol 170: 212-217. doi:10.1016/j.vetpar.2010.02.010. PubMed: 20299152. 20299152 10.1016/j.vetpar.2010.02.010

[66] Bounias M, Kruk I, Nectoux M, Popeskovic D (1996) Toxicology of cupric salts on honeybees. V. Gluconate and sulfate action on gut alkaline and acid phosphatases. Ecotoxicol Environ Saf 35: 67-76. doi:10.1006/eesa.1996.0082. PubMed: 8930506. 8930506 10.1006/eesa.1996.0082

[67] http://www.r-project.org/.

[68] Cox DR (1972) Regression models and life tables. Biometrics 38: 67–77.

[69] de Groot AP (1953) Protein and amino acid requirements of the honey bee (*Apis mellifica* L.). Physiol Comp Oecol 3: . pp. 197-285.

[70] Bitondi MMG, Simões ZLP (1996) The relationship between level of pollen in the diet, vitellogenin and juvenile hormone titres in Africanized *Apis mellifera* workers. J Apic Res 35: 27-36.

[71] Hahn DA, Denlinger DL (2011) Energetics of insect diapause. Annu Rev Entomol 56: 103-121. doi:10.1146/annurev-ento-112408-085436. PubMed: 20690828. 20690828 10.1146/annurev-ento-112408-085436

[72] Toth AL, Kantarovich S, Meisel AF, Robinson GE (2005) Nutritional status influences socially regulated foraging ontogeny in honey bees. J Exp Biol 208: 4641-4649. doi:10.1242/jeb.01956. PubMed: 16326945. 16326945 10.1242/jeb.01956

[73] Herbert EW, Shimanuki H, Shasha BS (1980) Brood Rearing and Food-Consumption by Honeybee Colonies Fed Pollen Substitutes Supplemented with Starch-Encapsulated Pollen Extracts. J Apicult Res 19: 115-118.

[74] Tasei JN, Aupinel P (2008) Nutritive value of 15 single pollens and pollen mixes tested on larvae produced by bumblebee workers (*Bombus terrestris*, Hymenoptera: Apidae). Apidologie 39: 397-409. doi:10.1051/apido:2008017.

[75] Lee KP, Simpson SJ, Wilson K (2008) Dietary protein-quality influences melanization and immune function in an insect. Funct Ecol 22: 1052-1061. doi:10.1111/j.1365-2435.2008.01459.x.

[76] Klemola N, Klemola T, Rantala MJ, Ruuhola T (2007) Natural host-plant quality affects immune defence of an insect herbivore. Entomol Exp Appl 123: 167-176. doi:10.1111/j.1570-7458.2007.00533.x.

[77] Lee KP, Cory JS, Wilson K, Raubenheimer D, Simpson SJ (2006) Flexible diet choice offsets protein costs of pathogen resistance in a caterpillar. Proc Biol Sci 273: 823-829. doi:10.1098/rspb.2005.3385. PubMed: 16618675. 16618675 10.1098/rspb.2005.3385 PMC1560230

[78] Brakefield PM (1987) Industrial melanism: Do we have the answers? Trends Ecol Evol 2: 117-122. doi:10.1016/0169-5347(87)90051-6. PubMed: 21227832. 21227832 10.1016/0169-5347(87)90051-6

[79] Campana BJ, Moeller FE (1977) Honey bees: preference for and nutritive value of pollen from 5 plant sources. J Econ Entomol 70: 39-41.

[80] Singh RP, Singh PN (1996) Amino acid and lipid spectra of larvae of honey bee (*Apis cerana* Fabr) feeding on mustard pollen. Apidologie 27: 21-28. doi:10.1051/apido:19960103.

[81] Foley K, Fazio G, Jensen AB, Hughes WOH (2012) Nutritional limitation and resistance to opportunistic *Aspergillus* parasites in honey bee larvae. J Invertebr Pathol 111: 68-73. doi:10.1016/j.jip.2012.06.006. PubMed: 22750047. 22750047 10.1016/j.jip.2012.06.006

[82] Eguchi M (1995) Alkaline phosphatase isozymes in insects and comparison with mammalian enzyme. Comp Biochem Physiol B Biochem Mol Biol 111: 151-162. doi:10.1016/0305-0491(94)00248-S. PubMed: 7599983. 7599983 10.1016/0305-0491(94)00248-s

[83] Le Conte Y, Navajas M (2008) Climate change: impact on honey bee populations and diseases. Rev Sci Tech Off Int Epiz 27: 499-510. PubMed: 18819674. 18819674

