# Supplementary material for: Influence of Pollen Nutrition on Honey Bee Health: Do Pollen Quality and Diversity Matter?
Source: PLoS One. 2013 Aug 5;8(8):e72016. doi: 10.1371/journal.pone.0072016 (PMC3733843; doi:10.1371/journal.pone.0072016)
Supplement: Table S2 — Amino acids present in the different pollens. Their concentration is expressed in g/100 g of pollen. (DOCX) [file pone.0072016.s002.docx]

**Table S2**

| Amino-acids | *Cistus* | *Erica* | *Castanea* | *Rubus* |
| --- | --- | --- | --- | --- |
| Aspartic acid | 1.65 | 1.16 | 1.85 | 2.76 |
| Threonine | 0.47 | 0.82 | 0.87 | 0.94 |
| Serine | 0.56 | 0.9 | 1 | 1.1 |
| Glutamic acid | 1.1 | 2.09 | 2.29 | 2.35 |
| Proline | 2.25 | 1.29 | 2 | 1.58 |
| Glycine | 0.45 | 0.8 | 0.82 | 0.85 |
| Alanine | 0.6 | 0.98 | 1.07 | 1.17 |
| Valine | 0.54 | 1.01 | 1.05 | 1.14 |
| Cysteine | 0.16 | 0.23 | 0.36 | 0.31 |
| Methionine | 0.32 | 0.44 | 0.51 | 0.54 |
| Isoleucine | 0.45 | 0.8 | 0.84 | 0.91 |
| Leucine | 0.81 | 1.34 | 1.36 | 1.48 |
| Tyrosine | 0.3 | 0.57 | 0.58 | 0.59 |
| Phenylalanine | 0.48 | 0.9 | 0.89 | 0.99 |
| Lysine | 0.77 | 1.23 | 1.43 | 1.5 |
| Histidine | 0.37 | 0.44 | 0.47 | 0.46 |
| Arginine | 0.49 | 1.1 | 1.07 | 1.03 |
| Tryptophan | 0.14 | 0.21 | 0.23 | 0.27 |
