# Supplementary material for: Influence of Pollen Nutrition on Honey Bee Health: Do Pollen Quality and Diversity Matter?
Source: PLoS One. 2013 Aug 5;8(8):e72016. doi: 10.1371/journal.pone.0072016 (PMC3733843; doi:10.1371/journal.pone.0072016)
Supplement: Table S3 — Sugars present in the different pollens. Their quantity is expressed in mg per g of pollen. nd: not detected and nq: present but not quantifiable. (DOCX) [file pone.0072016.s003.docx]

**Table S3**

| Sugars | *Cistus* | *Erica* | *Castanea* | *Rubus* |
| --- | --- | --- | --- | --- |
| Trehalose | 0.48 | *nd* | 3.57 | *nd* |
| Glucose | 8.8 | 5.2 | 9.5 | 6.6 |
| Fructose | 6x10^-4^ | 3.7x10^-4^ | 7x10^-4^ | 8.5x10^-4^ |
| Saccharose | *nq* | 2.09 | 6.2 | 3.9 |
| Maltose | 1.3x10^-5^ | *nd* | 2.8x10^-5^ | 6.2x10^-6^ |
| Erlose | *nd* | *nd* | 0.47 | *nd* |
